# Machine learning-based predictive model for postoperative delirium of elderly patients with coronary heart disease undergoing non-cardiac surgery: a retrospective cohort study

**DOI:** 10.3389/fpsyt.2026.1780056

**Published:** 2026-03-27

**Authors:** Wenjie Kong, Jing Jiang, Yuanlong Wang, Jiayi Chen, Bin Wang, Kun Wang, Yizhi Liang, Jiahan Wang, Chuan Li, Yanan Lin, Hongyan Gong, Yongxin Liang, Yanlin Bi, Xu Lin

**Affiliations:** 1 The Second School of Clinical Medicine of Binzhou Medical University, Yantai, China; 2 Department of Anesthesiology, Qingdao Municipal Hospital, Qingdao, Shandong, China; 3 Department of Anesthesiology, Weifang Medical University, Weifang, China; 4 Department of Anesthesiology, Peking University People’s Hospital, Women and Children’s Hospital, Qingdao University, Qingdao, China

**Keywords:** coronary heart disease, delirium, machine learnings, prediction model, Shapley additive interpretation

## Abstract

**Background:**

Patients with advanced age and coronary heart disease (CHD) are at significantly increased risk for postoperative delirium (POD). However, there is no method to predict POD in elderly patients with CHD.

**Methods:**

Date from elderly patients with CHD who underwent non-cardiac surgery was collected. The dataset is subdivided into training and validation sets at a ratio of 7:3. Boruta algorithm, least absolute shrinkage and selection operator (LASSO) regression and multiple logistic regression analysis were used to select features. Machine learning method was used to construct a model for predicting the occurrence of POD. Receiver operating characteristic (ROC) curve, decision curve, calibration curve, specificity, sensitivity, accuracy, F1 score and Brier score were used to compare the predictive performance of these machine learning models, and the interpretability of the models was evaluated by Shapley additive interpretation (SHAP).

**Results:**

A total of 861 patients were included in the study. The incidence of POD was 16.6% (143/861). Seven key features were identified. Ten machine learning models were constructed. Among the models, gradient boosting model (GBM) performed better. The area under the ROC curve (AUC) is 0.856 (95% confidence interval [CI]: 0.796-0.916). The decision curve, calibration curve, specificity, sensitivity, accuracy, F1 score and Brier score were also relatively good. SHAP plots of GBM showed that Clinical Frailty Scale (CFS) grade, Mini-mental State Examination (MMSE) score, and Athens Insomnia Scale (AIS) score were significant predictors of POD in elderly CHD patients, and an easy-to-use calculator for predicting the risk of POD was developed based on the GBM model.

**Conclusion:**

This study developed a reliable GBM model for predicting the occurrence of POD in elderly patients with CHD. Higher CFS grade, lower MMSE score and higher AIS score significantly enhanced the predictive ability of the model. External validation of our model is needed before it can be applied in a clinical setting.

**Trial registration:**

Registration number of the Chinese Clinical Trial Registry: ChiCTR2500097325, Registration Date: 17/02/2025.

## Highlights

• Ten machine learning models based on seven patient-relevant features, especially gradient boosting machine (GBM), performed well in predicting the occurrence of POD in elderly patients with CHD.
• The key features to explain the prediction of POD in elderly patients with CHD were CFS grade, MMSE score and AIS score.
• The higher the CFS grade, the lower the MMSE score, and the higher the AIS score, the stronger the explanatory power of the model.
• We built an online calculator, used to predict the elderly patients with CHD the incidence of POD(https://predictionmodel99.shinyapps.io/webbb/).

## Introduction

Postoperative delirium (POD) is a complex syndrome common in elderly patients after surgery, characterized by acute and fluctuating impairment of consciousness, attention, cognition, and perception (1–3), which increases the incidence of postoperative falls, prolongs hospital stays, and is also associated with poor functional recovery, long-term dementia, and increased mortality (4–6). In the face of the medical, social and economic problems caused by POD, it is of great practical significance to identify the occurrence of POD as early as possible and formulate reasonable and effective early warning measures.

Coronary heart disease (CHD) is a global problem. It is caused by narrowing or obstruction of the coronary artery, which leads to insufficient blood supply and hypoxia to the heart muscle (7, 8). From 1990 to 2019, the incidence and mortality of CHD have continued to increase globally, especially in low and middle-income countries and older populations (9). Previous studies have shown that age is an independent risk factor for POD (1), and CHD is also significantly associated with postoperative cognitive impairment (10). The coexistence of these two diseases may have an additive adverse effect on the occurrence of POD. Given the large number of elderly CHD patients at high risk for POD, earlier and more effective strategies are needed to identify and reduce the risk.

In recent years, the application of artificial intelligence (AI) technology in the field of medical care has become increasingly prevalent. In addition to assisting in the diagnosis and treatment of diseases, the technology has also expanded to the field of predicting clinical outcomes (11, 12). Machine learning, as a significant subfield of AI, has the ability to enable systems to independently learn and identify patterns from data, and to make corresponding predictions (13). In comparison to traditional prediction methods, machine learning has the capacity to analyze clinical indicators, population statistics, and complex non-linear relationships between variables, thereby aiming to achieve more precise risk stratification. Additionally, this technology can utilize updated data to continuously optimize prediction results (14, 15). Previous studies have explored the application of machine learning in predicting POD in intensive care unit (ICU) patients or elderly hospitalized populations (16, 17). However, research on predicting POD in elderly patients with CHD is still limited. For personalized health management of the elderly with CHD, it is necessary to establish a prediction model with high accuracy and good interpretability to promote the realization of precision medicine.

In view of these needs, this study aimed to explore the development and interpretation of machine learning models for predicting the occurrence of POD in elderly patients with CHD.

## Materials and methods

### Research methods

This is a single-center, observational, retrospective cohort study involving elderly patients with CHD who underwent non-cardiac surgery in Qingdao Municipal Hospital from January 2022 to December 2023. This trial was performed in accordance with the Declaration of Helsinki principles. The study protocol was approved by the Ethics Committee of Qingdao Municipal Hospital (Approval Number:2024-KY-095) and registered in the Chinese Clinical Trial Registry(Registration Number: ChiCTR2500097325, Registration Date: 17/02/2025).

The inclusion criteria were CHD patients aged ≥65 years undergoing non-cardiac surgery. The main exclusion criteria were: (1) Preoperative Mini-mental State Examination (MMSE) score < 23; (2) Emergency surgery or major surgery such as cardiovascular surgery within one month; (3) Central nervous system infection, head trauma, epilepsy, multiple sclerosis and other major nervous system diseases; (4) Heart function grade IV, severe liver and kidney dysfunction, hemorrhagic disease; (5) Mental or conscious disturbance; (6) Drug abuse, psychotropic drug abuse, and long-term use of steroids and hormones. In addition, patients with missing key data, those whose signatures were rejected for relevant documents related to medical research, those who were lost to follow-up, and those who died after surgery were excluded. Baseline characteristics, including demographic data, past medical history, and preoperative and postoperative visit scores, were recorded.

This study involved a variety of machine learning algorithms, feature selection methods, and assessment scales. These terms are explained in the Glossary at the end of the article, as well as when they first appear.

### Follow-up and end points

Anesthesiologists obtained basic information about patients from medical records and preoperative visits. General patient information and preoperative indicators: Gender, age, height, weight, anesthesia method, past medical history (hypertension, diabetes, CHD), smoking history, drinking history, COVID-19 infection history, years of education, Mini-mental State Examination (MMSE) score, Pittsburgh Sleep Quality Index (PSQI, a sleep quality assessment tool) score, Athens Insomnia Scale (AIS, an insomnia assessment scale) score, Mini nutritional assessment score (MNA, nutritional status assessment scale; MNA≥24: good nutritional status; 17≤MNA < 24: at risk of malnutrition; MNA < 17: confirmed malnutrition), The FRAIL Scale score (a frailty screening scale), Clinical Frailty Scale (CFS, a frailty screening scale) grade, the presence or absence of subjective cognitive decline, physical activity (PA) or not, metabolic equivalent (MET) (18); Intraoperative indicators: intraoperative fluid, estimated blood loss, operation duration, anesthesia duration; Postoperative indicators: Numerical Rating Scale (NRS) score on 1 day after surgery. The positive criteria of subjective cognitive decline is that the patient complains of recent persistent cognitive decline (especially memory) and worries about it, but has normal performance on conventional objective cognitive tests. The PA was defined as the frequency of physical activity more than 3 times per week, the duration of each activity more than 30 minutes, and the intensity of each activity more than moderate.

Delirium was assessed twice after surgery by another group of anesthesiologists from 9 am to 10 am and 2 PM to 3 PM daily from 1 to 7 days after surgery (or before discharge). Confusion Assessment Method (CAM) was used to assess delirium. All the anesthesiologists who participated in CAM assessment received standardized training, including theoretical lectures, video case exercises and supervised bedside assessments. Before the start of the study, 20 patients were selected for independent two-person evaluation, the Cohen’s kappa coefficient was 0.92, indicating excellent inter-rater reliability. At the same time, to reduce missed diagnoses, assessors performed daily structured chart reviews to screen for clues to delirium. Suspicious cases were adjudicated by the principal investigator to ensure the completeness of the delirium diagnosis. Patients with a positive assessment were assigned to the POD group, and those with a negative assessment were assigned to the non-postoperative delirium (NPOD) group.

### Definition

The diagnosis of CHD was confirmed by both attending physicians and board-certified cardiologists through comprehensive evaluation including clinical history, coronary angiography, and the fulfillment of any of diagnostic criteria: (1) At least one major coronary artery has a lumen diameter stenosis of ≥50% as confirmed by coronary angiography; (2) There is a clear history of hospitalization for acute myocardial infarction, and the diagnosis conforms to the Fourth Universal Definition of Myocardial Infarction; (3) There is a history of percutaneous coronary intervention or coronary artery bypass grafting. All diagnoses were verified by reviewing the hospital’s electronic medical records and imaging reports.

### Machine learning to analyze the participants

Our machine learning analysis aimed to develop predictive models based on clinical features and scores to straticategorize people at high risk for POD by: (1) classify participants into two categories: those who developed POD during follow-up and those who did not; (2) to maintain objectivity and ensure the reproducibility of the study, patients were randomly assigned into two cohorts using a random seed: a training cohort comprising 70% of the patients and a validation cohort comprising the remaining 30%. (3) the training cohort is used for developing and optimizing predictive models, while the validation cohort serves as an independent dataset to evaluate the predictive performance of these models. (4) evaluate the predictive performance of the machine learning model; And (5) visualize machine learning results to make them easy to understand.

### Data processing

All variables undergo data preprocessing, which is represented as continuous variables or categorical variables, to meet the requirements of machine learning data. Variables with more than 20% missing values were eliminated, and the remaining missing values were handled using multiple imputation methods.

### Feature selection in machine learning analysis

In the designed report form, a total of 25 features were included. Boruta algorithm, least absolute shrinkage and selection operator (LASSO) regression and multiple logistic regression analysis were used to select features for machine learning analysis. Boruta algorithm is a feature ranking and selection algorithm based on random forest algorithm (19), and LASSO regression is a regularization method that uses L1 norm for penalty (20). Logistic analysis is a classical method of variable selection based on p-value, and variables with p-value less than 0.05 are considered statistically significant. The final features for the machine learning analysis were determined on the basis of these three screening methods, with simultaneous consideration of clinical importance.

### Construction and performance evaluation of machine learning models

In this study, based on the diversity and expressiveness of the model, the ability to deal with different types of data, the handling of overfitting, and the practical application, we built ten machine learning models. These models include logistic regression (LR), support vector machine (SVM), gradient boosting model (GBM), neural network (NN), random forest (RF), extreme gradient boosting (Xgboost), K-nearest neighbor (KNN), Adaptive Boosting (AdaBoost), Light Gradient-Boosting model (LightGBM), Categorical Boosting (CatBoost). The construction and evaluation of the model are divided into two stages: hyperparameter selection and performance validation. In the hyperparameter selection stage, we use grid search combined with 10-fold cross-validation, aiming to maximize the average AUC, to determine the optimal parameter combination from the parameter grid. Subsequently, in the performance validation stage, to obtain a robust estimate of the performance of the optimal model, we fix its hyperparameters and use 10-fold cross-validation repeated 5 times. The final reported AUC value is the average of all 50 test folds’ results from these 5 repetitions. All random processes are controlled by setting a fixed random seed (42) to ensure the reproducibility of the experiment.

The predictive performance was strictly evaluated in validation queue. The model’s predictive accuracy, robustness and clinical applicability in predicting mortality were comprehensively evaluated using AUC, AUC 95% Confidence Interval (CI), accuracy, specificity, sensitivity, F1-score, Brier score and decision curve.

### Model interpretability

The interpretability of a model is done through Shapley Additive Interpretation (SHAP) (21), a method used to interpret the output of a machine learning model through Shapley values, which measure the importance, dependence, and interaction between different variables of global and local features and visualize the characteristics of a given observation.

### Statistical analysis

To test for normality, Kolmogorov-Smirnov tests were used for all continuous variables. When continuous data were normally distributed, the independent sample t-test was used to compare the differences between groups. Continuous variables non-normal distribution characteristics, the median and interquartile range (IQR), and comparing the mann - Whitney U test. The classification variables to count and percentage, and chi-square test were compared.

The partitioning of the entire dataset, feature selection for Boruta and LASSO regression, machine learning algorithms, prediction performance evaluation, and SHAP visualization of the GBM model were done by R software (version 4.4.2, Austria). The baseline data analysis and multiple logistic regression analysis were performed by SPSS 25.0.0 software. Two-sided P values of less than 0.05 were considered to indicate statistical significance.

## Results

### Baseline data analysis

**Figure 1:**
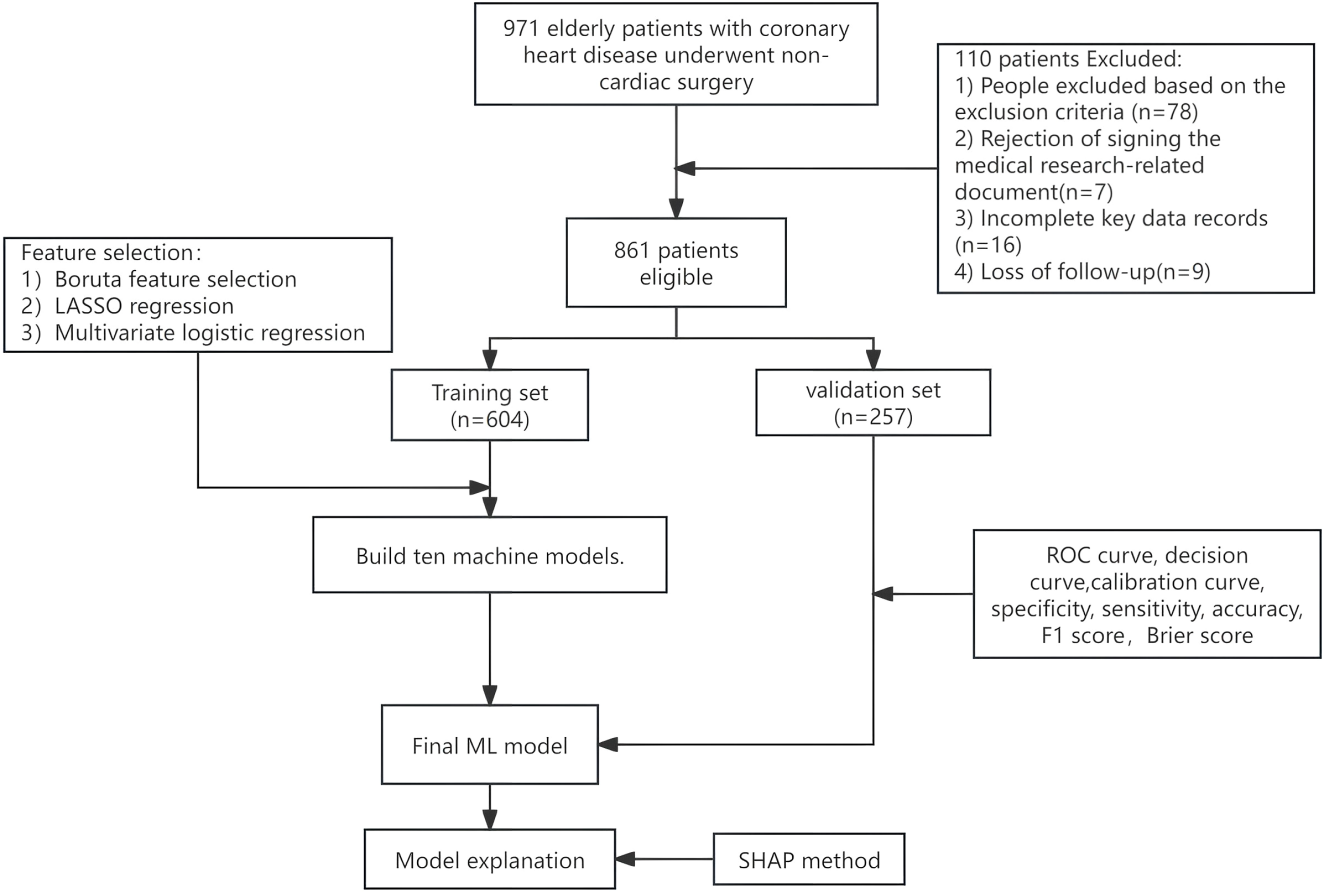
Flow diagram.

After screening out elderly patients with CHD (n = 976), and after applying the exclusion criteria, a total of 861 cases were finally included in the analysis. **Figure 1** presents a comprehensive flowchart detailing the enrollment process and outlining the entire study design.

The incidence of POD was 16.6% (143/861). There were differences between the POD group and the NPOD group in terms of diabetes history, the presence of subjective cognitive decline, MNA (nutritional status assessment scale) classification, PA, CFS (frailty screening scale) grade, MMSE score, age, AIS (insomnia assessment scale) score, years of education, PSQI (sleep quality assessment scale) score, The FRAIL Scale score (frailty screening scale) and NRS score. (P < 0.05). (**Table 1**).

**Table 1:** Clinical characteristics of participants.

Variable	All participants (n=861)	POD (n=143)	NPOD (n=718)	*P*
Female[n (%)]	367 (42.6)	61 (42.7)	306 (42.6)	0.993
Height[cm,M (IQR)]	166 (160-172)	165 (160-172)	166 (160-172)	0.416
Weight[kg,M (IQR)]	68 (60-75)	67 (60-75)	68 (60-75)	0.804
Anesthesia method[n (%)]				0.356
General anesthesia	546 (63.4)	97 (67.8)	449 (62.5)	
Spinal anesthesia	311 (36.1)	46 (32.2)	265 (36.9)	
Nerve block	4 (0.5)	0 (0)	4 (0.6)	
Hypertension = [n (%)]	325 (37.7)	99 (69.2)	437 (60.9)	0.059
Diabetes [n (%)]	579 (67.2)	65 (45.5)	217 (30.2)	<0.001***
Subjective cognitive decline [n (%)]	400 (46.5)	90 (62.9)	310 (43.2)	<0.001***
MNA [n (%)]				<0.001***
Good nutritional status	314 (36.5)	37 (25.9)	277 (38.6)	
Risk of malnutrition	358 (41.6)	47 (32.9)	311 (43.3)	
Definite malnutrition	189 (22.0)	59 (41.3)	130 (18.1)	
Smoking [n (%)]	205 (23.8)	28 (19.6)	177 (24.7)	0.194
Alcohol [n (%)]	165 (19.2)	23 (16.1)	142 (19.8)	0.306
COVID_19 [n (%)]	832 (96.6)	138 (96.5)	694 (96.7)	0.926
PA [n (%)]	331 (38.4)	21 (14.7)	310 (43.2)	<0.001***
CFS grade[n (%)])				<0.001***
I	396 (46)	14 (9.8)	382 (53.2)	
II	204 (23.7)	25 (17.5)	179 (24.9)	
III	115 (13.4)	23 (16.1)	92 (12.8)	
IV	96 (11.1)	53 (37.1)	43 (6.0)	
V	42 (4.9)	25 (17.5)	17 (2.4)	
VI	7 (0.8)	3 (2.1)	4 (0.6)	
VII	1 (0.1)	0 (0)	1 (0.1)	
MMSE [scores,M (IQR)]	27 (25-28)	25 (24-26)	27 (26-28)	<0.001***
Age [year,M (IQR)]	75 (71-81)	83 (77-88)	74 (70-79)	<0.001***
AIS [scores,M (IQR)]	3 (2-5)	4 (1-5)	3 (2-5)	0.029*
Education [year,M (IQR)]	9 (5-9)	6 (5-9)	9 (5-10)	0.007**
PSQI [scores,M (IQR)]	7 (4-10)	8 (4-12)	6 (4-10)	0.015*
The FRAIL Scale [scores,M (IQR)]	1 (0-2)	2 (1-3)	1 (0-2)	<0.001***
MET [M (IQR)]	3 (3-4)	3 (2-4)	3 (3-4)	0.131
Duration of surgery[h,M (IQR)]	2.08 (1.25-3.46)	2.08 (1.00-3.25)	2.08 (1.25-3.42)	0.213
Duration of anesthesia[h,M (IQR)]	3.00 (1.83-4.42)	2.75 (1.67-4.25)	3.00 (1.92-4.42)	0.119
Intraoperative fluid[ml,M (IQR)]	1100 (1000-2100)	1100 (1000-2100)	1300 (1000-2100)	0.793
Estimated blood loss[ml,M (IQR)]	20 (5-50)	20 (5-50)	20 (5-50)	0.515
NRS [scores,M (IQR)]	2 (1-4)	3 (2-4)	2 (1-4)	0.001**

MMSE, Mini-mental State Examination; PA, physical activity; PSQI, Pittsburgh Sleep Quality Index; AIS, Athens Insomnia Scale; MNA, Mini nutritional assessment; CFS, Clinical Frailty scale; MET, Metabolic Equivalent; NRS, Numerical Rating Scale.

*P-Value < 0.05, **P-Value < 0.01, ***P-Value < 0.001.

### Feature selection (for machine learning)

**Figure 2:**
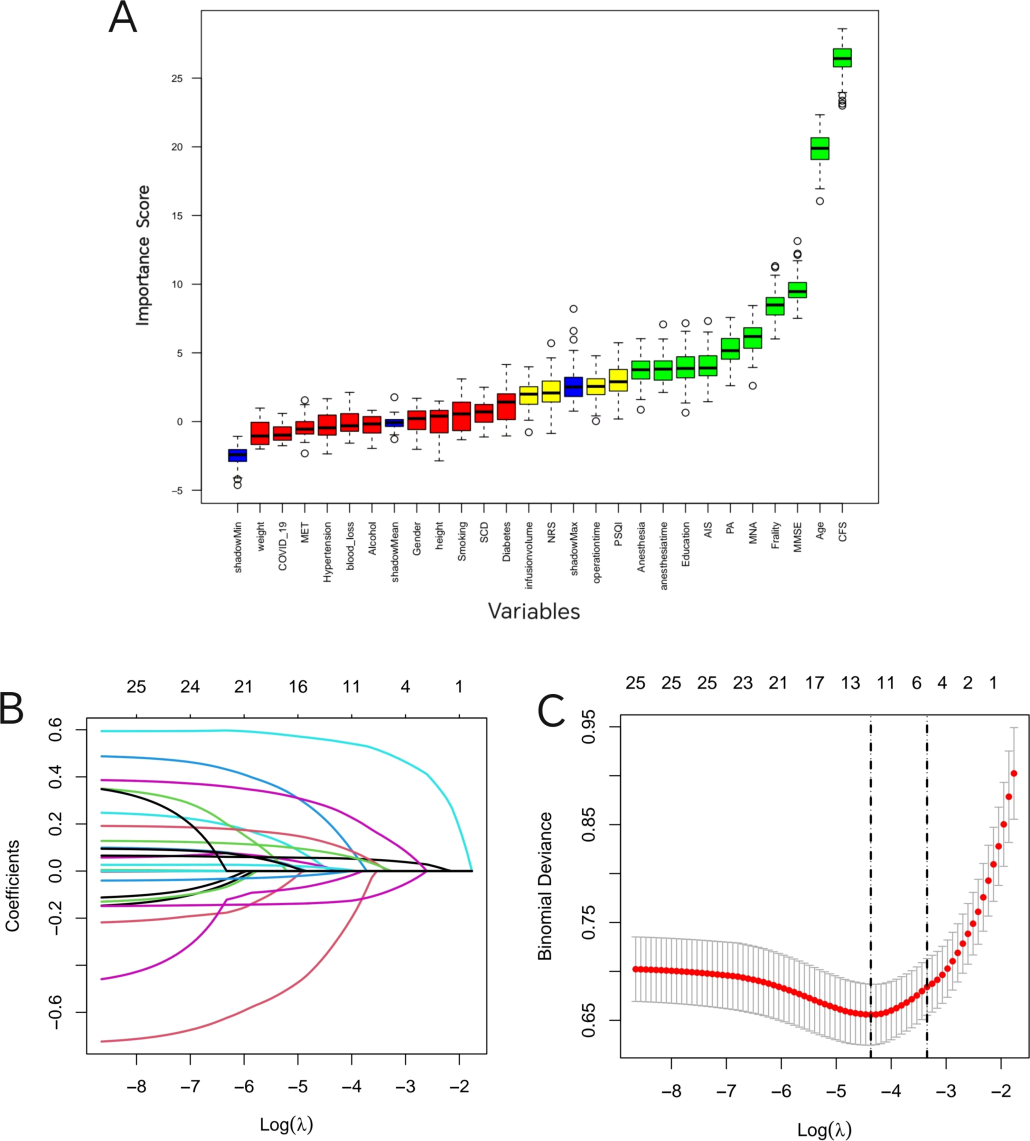
Feature selection based on Boruta and LASSO regression. **(A)** The blue curve represents the minimum, average and maximum shadow scores. Variables with green box plots are important variables, yellow ones are tentatively important variables, and red ones are excluded variables; **(B)** The correlation between the L1 norm and different coefficients in LASSO regression. The L1 norm is the regularization term of LASSO; **(C)** The correlation between lambda and binomial deviance. There are two dashed lines in the figure. The left dashed line indicates the minimum mean square error, and the right dashed line indicates the position one standard error away from the minimum mean square error. CFS, Clinical Frailty scale; MMSE, Mini-mental State Examination; AIS, Athens Insomnia Scale; Frailty, The FRAIL Scale score; SCDS, Subjective Cognitive Decline; PA, physical activity; PSQI, Pittsburgh Sleep Quality Index; MNA, Mini nutritional assessment.

Three methods are used to filter features. Boruta feature selection confirmed 12 features (anesthesia method, MNA classification, PA, CFS grade, MMSE score, age, AIS score, years of education, PSQI score, The FRAIL Scale score, operation duration, anesthesia duration), 12 features (anesthesia method, MNA classification, PA, CFS grade, MMSE score, age, NRS score, AIS score, PSQI score, The FRAIL Scale score, anesthesia duration) were selected for LASSO regression. 10 potential predictive features (anesthesia method, MNA classification, PA, CFS grade, MMSE score, age, AIS score, years of education, PSQI score, The FRAIL Scale score, anesthesia duration) were selected by intersection of these two methods and included in the multiple logistic regression. 7 features (CFS grade, MMSE score, age, AIS score, anesthesia method, The FRAIL Scale score, MNA classification) were finally determined by multiple logistic regression analysis (**Figure 2**, **Table 2**).

**Table 2:** The results of multiple logistic regression.

Variable	*B*	*OR*	95%*CI*	*P*
Anesthesia method	-0.717	0.488	0.265-0.898	0.021*
PA	0.409	1.506	0.760-2.984	0.241
CFS grade	0.694	2.002	1.619-2.474	<0.001***
MMSE	-0.152	0.859	0.747-0.988	0.033*
Age	0.042	1.043	1.001-1.087	0.047*
AIS	0.165	1.179	1.095-1.269	<0.001***
PSQI	0.033	1.033	0.961-1.111	0.373
The FRAIL Scale score	0.255	1.290	1.019-1.635	0.035*
Duration of anesthesia	0.001	1.001	0.872-1.150	0.986
MNA	0.405	1.499	1.047-2.146	0.027*

PA, physical activity; CFS, Clinical Frailty scale; MMSE, Mini-mental State Examination; AIS, Athens Insomnia Scale; PSQI, Pittsburgh Sleep Quality Index; MNA, Mini nutritional assessment;.

*P-Value < 0.05, **P-Value < 0.01, ***P-Value < 0.001.

### Machine learning model evaluation

**Figure 3:**
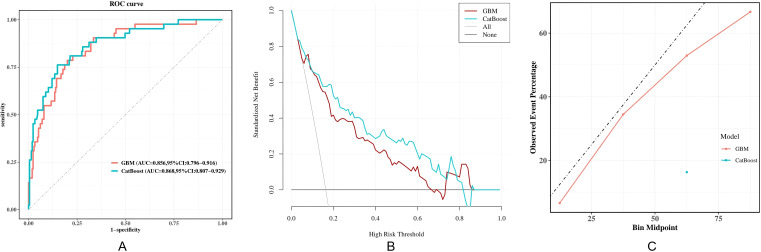
The receiver operating characteristic curves, decision curves and calibration curves of the two superior models. **(A)** ROC curve of the validation set; **(B)** Decision curve of the validation set; **(C)** Calibration curve of the validation set. Comparison of curves for the 10 models is shown in **Supplementary Figure 1**. GBM, gradient boosting machine; CatBoost, Categorical Boosting; AUC, Area under the ROC curve.

Among the ten models, CatBoost (AUC = 0.868, 95% CI:0.807-0.929) and GBM (AUC = 0.856, 95% CI:0.796-0.916) perform better. However, the calibration curve and Brier score (0.277) indicated that the CatBoost had poor calibration. So the GBM model is the best model. The GBM also showed balanced accuracy (79.8%), sensitivity (78.6%), Specificity(80%), and F1 value (0.559), with a high net benefit in the decision curve analysis (**Figure 3**; **Table 3**). Comparison of curves for the 10 models is shown in **Supplementary Figure 1**.

**Table 3:** Evaluate the performance of 10 machine learning models in the validation dataset.

Model	AUC	Accuracy	Sensitivity	Specificity	F1 score	Brier score
LR	0.843 (0.772-0.914)	0.798	0.786	0.8	0.559	0.099
SVM	0.821 (0.745-0.897)	0.759	0.833	0.744	0.53	0.109
GBM	0.856 (0.796-0.916)	0.798	0.786	0.8	0.559	0.102
NN	0.854 (0.791-0.918)	0.743	0.881	0.716	0.529	0.100
RF	0.832 (0.763-0.901)	0.716	0.905	0.679	0.51	0.106
Xgboost	0.784 (0.715-0.853)	0.786	0.667	0.809	0.505	0.131
KNN	0.820 (0.746-0.893)	0.829	0.667	0.86	0.56	0.107
Adaboost	0.785 (0.711-0.859)	0.755	0.786	0.749	0.512	0.123
LightGBM	0.753 (0.662-0.845)	0.755	0.714	0.763	0.488	0.143
CatBoost	0.868 (0.807-0.929)	0.767	0.881	0.744	0.552	0.277

LR, logistic regression; SVM, support vector machine; GBM, gradient boosting machine; XGBoost, extreme gradient boosting; NN, neural network; RF, random forest; KNN,K-nearest neighbor; AdaBoost, Adaptive Boosting; LightGBM, Light Gradient-Boosting Machine; CatBoost, Categorical Boosting; AUC, Area under the ROC curve.

### Model interpretability and online predictions

**Figure 4:**
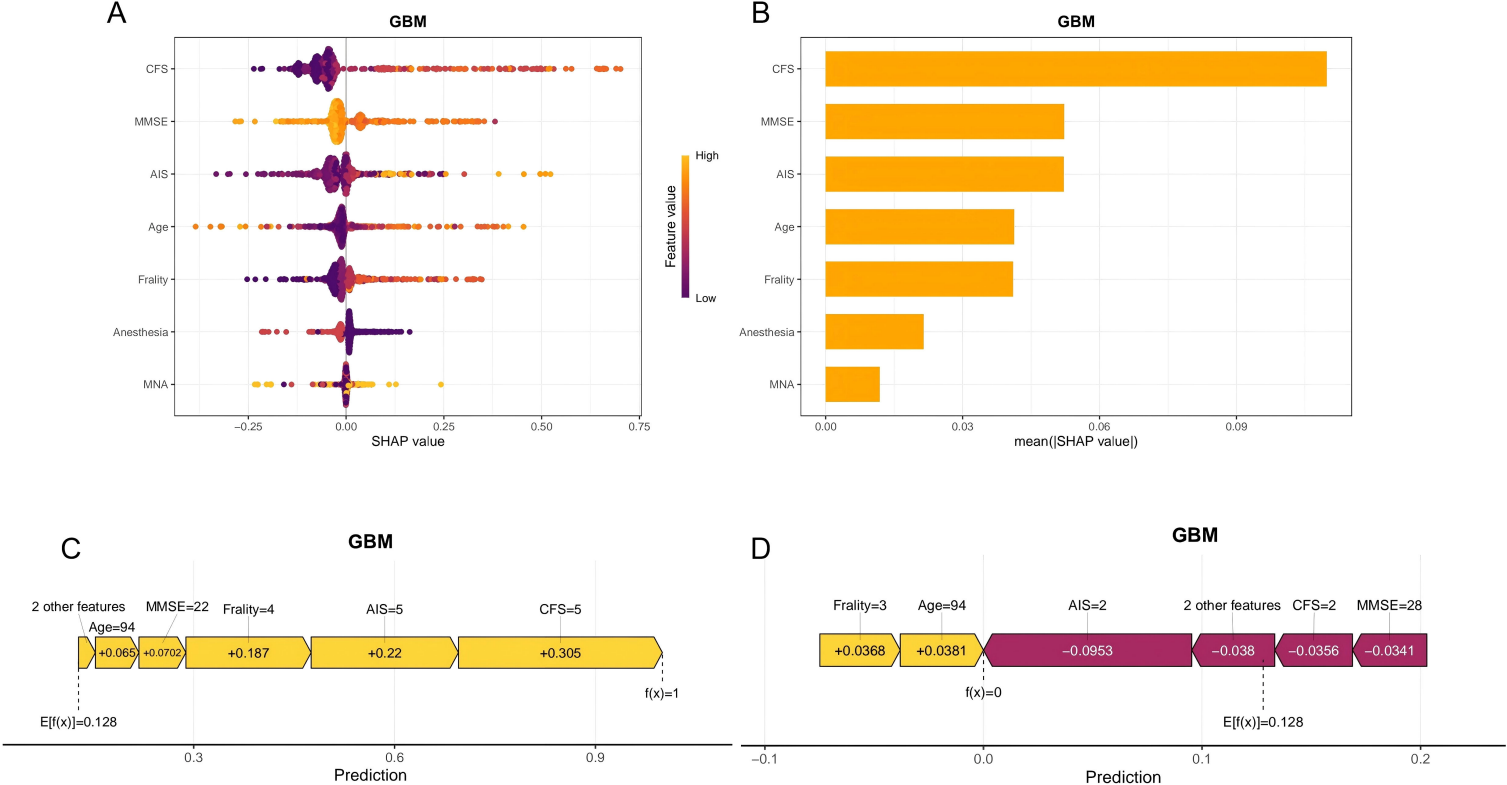
Visual explanation of the delirium model for elderly patients with CHD based on GBM. **(A)** The Shapley Additive explanation; **(B)** Feature importance scores. Force plot shows the contribution of each feature to the prediction result of using the GBM model. **(C)** One sample with a positive outcome prediction; **(D)** One sample with a negative outcome prediction. Orange bars indicate features that contribute positively to the prediction, while purple bars indicate negative contributions. Feature values are shown alongside their SHAP values.

Since the GBM model had the best predictive performance among the ten machine learning models, this model was chosen to explain its output results. The seven most important features were CFS grade, MMSE score, age, AIS score, anesthesia method, The FRAIL Scale score, MNA in turn. SHAP values provide more insight into how GBM models predict outcomes. The feature importance summarized by the SHAP summary plot is shown in **Figure 4**. The three most important features were CFS grade, MMSE score, and AIS score. The increase of CFS grade, the decrease of MMSE score, and the increase of AIS score were associated with the increased interpretability of the model. To demonstrate the SHAP calculation process, representative samples were selected: one with a positive outcome prediction (**Figure 4C**) and one with a negative outcome prediction (**Figure 4D**). The GBM-derived SHAP plot illustrates feature contributions for two patients. Orange/purple bars denote positive/negative impacts, with actual values alongside SHAP values.

**Figure 5:**
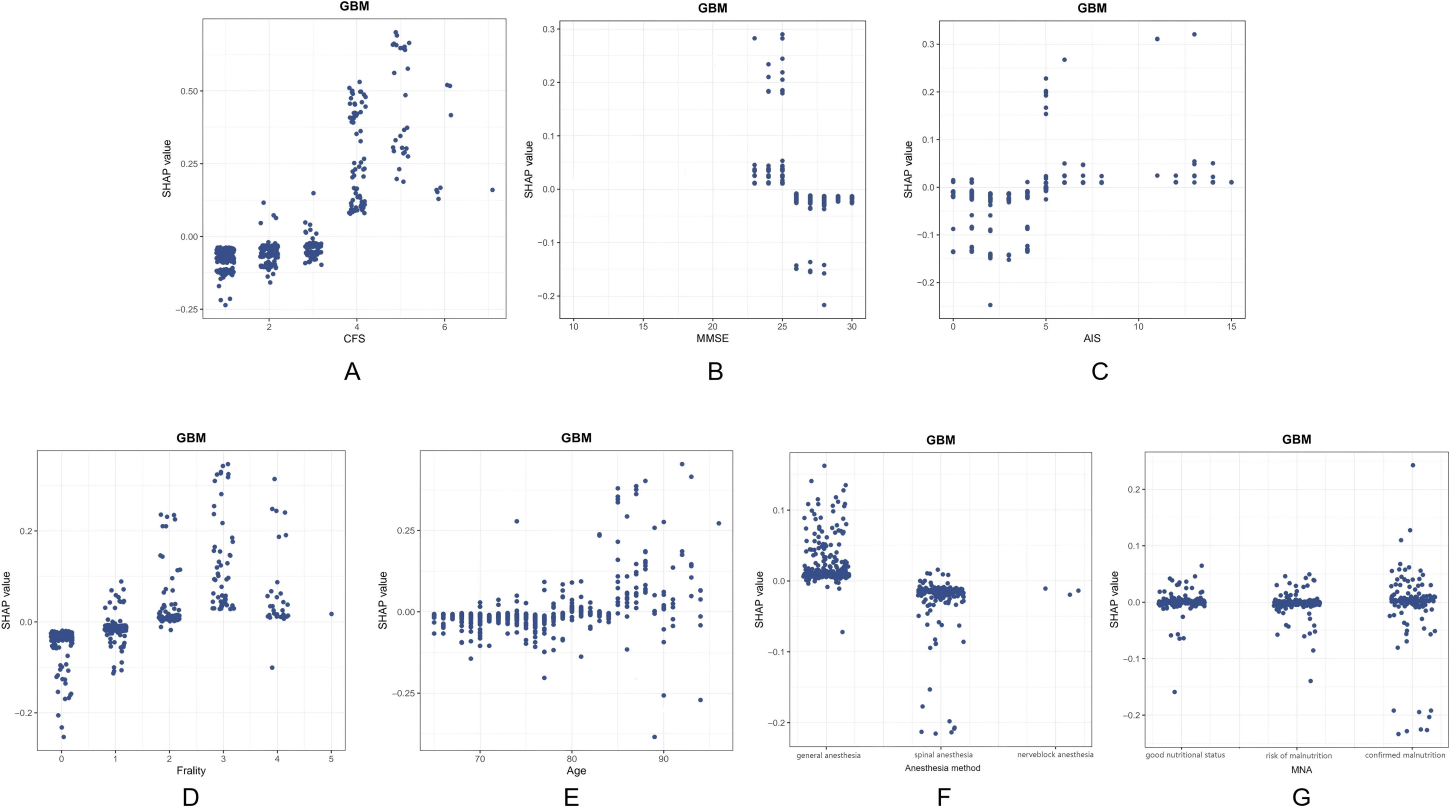
Seven important feature SHAP dependence plots. **(A)** CFS, Clinical Frailty scale; **(B)** MMSE, Mini-mental StateExamination; **(C)** AIS, Athens Insomnia Scale; **(D)** Age; **(E)** Frailty, The FRAIL Scale score; **(F)** anesthesia method; **(G)** MNA, Mini nutritional assessment. SHAP dependence plots show how individual features affect the output of the GBM model. A positive SHAP value for a specific feature indicates an increased risk of delirium.

We also quantitatively visualized the relationship between the main risk features and the outcome. The SHAP dependence plots show the seven important features. This indicates that higher CFS grade, AIS score, The FRAIL Scale score, and age, a lower MMSE score, the method of general anesthesia, as well as definite malnutrition increase the risk of POD. At the same time, the cutoff values of each feature can be determined to distinguish between high-risk and low-risk groups (**Figure 5**).

**Figure 6:**
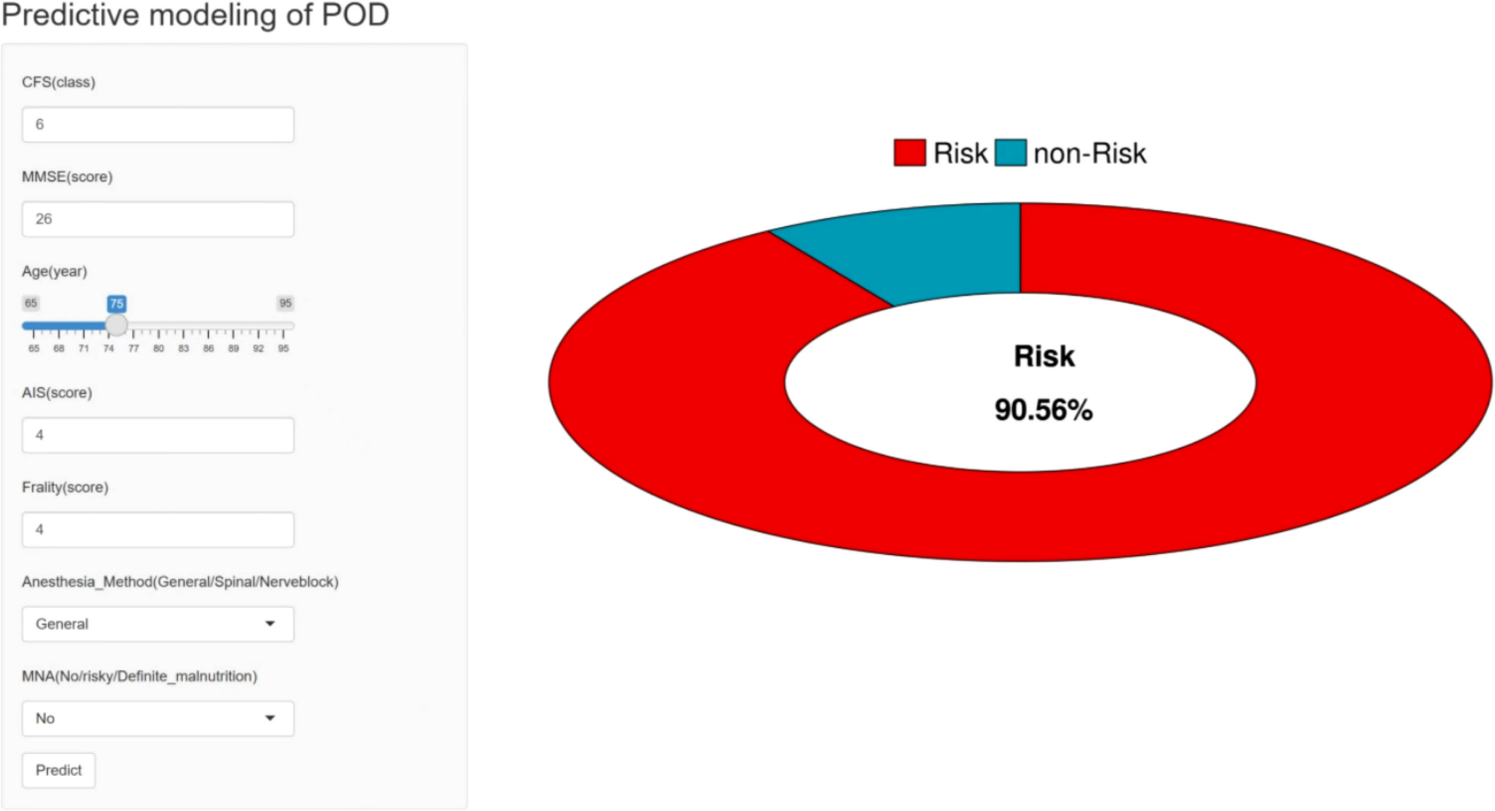
Online calculator.

In order to promote preoperative clinical applicability of the model, we built an easy to use online calculator, used to predict the old patients with CHD the incidence of POD, and offered on https://predictionmodel99.shinyapps.io/webbb/ (**Figure 6**).

## Discussion

The results of this large retrospective cohort study of patients with CHD showed that (1) ten machine learning models based on seven patient-relevant features, especially gradient boosting machine (GBM), performed well in predicting the occurrence of POD in elderly patients with CHD. (2) The key features to explain the prediction of POD in elderly patients with CHD were CFS grade, MMSE score and AIS score; (3) The higher the CFS grade, the lower the MMSE score, and the higher the AIS score, the stronger the explanatory power of the model.

POD is an acute but transient neurocognitive disorder that usually occurs within one week after surgery. The incidence of POD in elderly patients undergoing elective noncardiac surgery is about 12-14% (1, 22, 23). The incidence of POD in this study was 16.6%, which is slightly higher than that in previous studies. Therefore, the co-existence of advanced age and CHD partly contributes to the occurrence of POD. However, the mechanism of POD is still unclear. Multi-factorial and interdependent mechanisms may underlie POD development (24). At present, it is believed that POD may be related to systemic stress inflammatory response, Aβ deposition and tau protein phosphorylation, blood-brain barrier damage, transmitter and receptor imbalance (25, 26). Although the mechanism of POD remains unclear, we hope to predict the occurrence of POD in advance through some clinical indicators, thereby strengthening the management of patients at high risk of POD.

CHD is a chronic disease, and this disease may last for years or even decades. On the basis of long-term immune response and atherosclerosis, patients with CHD will have varying degrees of blood-brain barrier damage (27). Surgical trauma is a process that promotes stress, leading to the release of a large number of inflammatory factors. These inflammatory factors penetrate the blood-brain barrier and cause nerve cell damage and neuroinflammation, which may leads to the occurrence of POD. Data from a large occupational cohort study in the United Kingdom showed a clear association between CHD and cognition (28). Several vascular risk factors and measures of vascular disease have also been linked to cognitive impairment and dementia (29–32). The atherosclerotic process and associated hypoperfusion are thought to be responsible for this association. Our previous research also found that a higher cardiovascular score is an independent risk factor for POD (30). Therefore, we chose elderly patients with CHD as the research subjects.

In this study, machine learning method was used to construct a prediction model. The performance of machine learning algorithms largely depends on the quality of the available data. In this study, we first use three commonly used and classical feature selection methods to select feature variables for machine learning. Boruta can make up for the disadvantage that Lasso may miss important nonlinear relationships, Lasso can make up for Boruta’s insensitivity to collinearity, and multivariate regression provides a traditional statistical inference framework. Multi-factor regression provides a traditional statistical inference framework, which is easier to be accepted in academic aspects. This shows that despite the different principles of these three feature selection methods, the results are robust. Common features of these methods include these parameters (i.e. CFS grade, MMSE score, age, AIS score, anesthesia method, The FRAIL Scale score, MNA classification).

A variety of machine learning models and algorithms exist, each with its own advantages. For example, LR models are easy to implement, perform well on low-dimensional data, and are very efficient for linear data. However, it is unable to capture complex nonlinear patterns among complex variables (33). RF model performs well in processing large-scale datasets, and can achieve high accuracy through multiple decision trees. However, it is prone to overfitting, which leads to poor generalization ability and lack of interpretability on new data (33). NN models can fit extremely complex nonlinear relationships and interaction effects, which can be applied to a variety of problems, such as image, speech, text, tabular data, etc. (34), but the interpretability is very poor, and it requires a large amount of data, and it is easy to overfit on small data sets (35). In our study, GBM model performed the best. GBM is the representative algorithm of the Bing family, which trains a series of decision trees serially, with each tree dedicated to fixing the errors of the previous one. The core idea is to gradually optimize the model along the gradient direction of the loss function (36). It can automatically capture complex nonlinear relationships and feature interactions (37). GBM is more friendly to large-scale data sets, and it has stronger fitting ability in theory, and often achieves higher accuracy than RF in practice.

Machine-learning algorithms operate like “black boxes,” so it may not be clear how they produce decision outputs (38). In recent years, interpretable machine learning models have been proposed to make black-box models more accurate and more interpretable through different methods, among which SHAP is a commonly used tool (39). SHAP is a game theory method based on Shapley value, which quantifies the contribution of each feature to the output result of machine learning model and explains how each feature affects the prediction probability. The final prediction result is obtained based on the sum of the average predicted value and all SHAP values. Therefore, SHAP not only contributes to the local interpretation of the model, but also contributes to the global interpretation, thus making the model more interpretable.

The SHAP algorithm indicated that the CFS grade was the most important predictor of POD. CFS, as a tool for assessing the degree of frailty in patients, can comprehensively capture their decreased physiological reserves and vulnerability to stress events. Frailty, as a clinical syndrome, is characterized by reduced physiological reserves, increased susceptibility to stressors, and is closely related to cognitive dysfunction (40). The chronic, low-grade systemic inflammation associated with frailty can damage the structure and function of the blood-brain barrier (41, 42). This allows peripheral inflammatory factors to cross into the central nervous system, especially under surgical stress. Once inside, these factors activate microglia, triggering neuroinflammation. This also disrupts the metabolism and balance of key neurotransmitters like dopamine and acetylcholine (43, 44), contributing to POD. At the same time, the combined effect of frailty and CHD impairs the hemodynamic stability of the brain. On the one hand, CHD itself implies the possible coexistence of cerebrovascular disease or systemic atherosclerosis. On the other hand, frail patients often have decreased cardiac reserve and weakened blood pressure regulation ability (45). The combination of these two factors severely impairs the brain’s self-regulation ability. During inevitable blood pressure fluctuations or hypoperfusion events in the perioperative period, the frail brain is unable to effectively maintain a constant blood flow, making it highly susceptible to transient cerebral ischemia and damaging the neuronal network. In addition, frailty patients often have a deficiency of neurotrophic factors (vitamin B12), which may further aggravate neurofunctional damage (46).

Two approaches were used to assess frailty status. The FRAIL Scale is a simple, symptom-based screening tool derived from comprehensive geriatric assessment data. It is fast, easy to use, and requires no special equipment, making it ideal for rapid screening on a large scale in the community or in primary care (47). The Clinical Frailty Scale is a global, clinical judgment-based grading tool. It requires assessors to rate the severity of frailty by taking into account a patient’s comorbidities, functional status, and cognitive ability (48). Compared with The FRAIL Scale, CFS has strong predictive value, global clinical judgment and good discrimination. The CFS has been shown to strongly predict important clinical outcomes such as in-hospital mortality, length of hospital stay, post-discharge placement needs (e.g., nursing homes), and 30-day readmission rates, this is particularly prominent among elderly patients admitted to acute care (49).

With the deepening of China’s healthcare system reform, how to optimize the allocation of medical resources in the face of the increasing number of elderly surgical patients has become a challenge. In this clinical study, we applied a machine learning model to predict the occurrence of POD in elderly patients with CHD. This novel approach identified several features that can be used to predict POD events, and we also further explored the critical values of features. CFS grade≥4, MMSE score <25, and AIS score≥5 led to an increased incidence of POD. In addition, a simple web-based calculator has also been constructed. The results of this study can provide evidence-based basis for this issue. Specifically, early identification of patients at high risk of POD can guide medical institutions to moderately tilt their resource allocation toward “preoperative and intraoperative optimization” and “postoperative brain health monitoring”. This policy orientation not only helps to improve the prognosis quality of individual patients through early intervention, but also can reduce long-term care dependence and long-term medical expenditures caused by POD at the social level, maximizing the social benefits of limited healthcare resources.

Some limitations of this study should be addressed. First, the sample size of older patients with POD in this large cohort of patients undergoing noncardiac surgery was relatively small, and the event rate was relatively low. Such imbalanced data pose a challenge for machine learning and can limit predictive performance. Second, SHAP, as a way to interpret the results of machine learning models, has its limitations. SHAP itself does not assume feature independence. However, in practical computations, for some models, the commonly used algorithms to estimate SHAP values often require approximations, and these approximation processes may introduce assumptions such as feature independence. When there are complex correlations among features, this may affect the accuracy of SHAP value interpretation. Third, this study may be subject to the potential risk of false positives due to multiple comparisons. Although LASSO regularization and 10-fold cross-validation were used to control for the risk of overfitting, these methods are not a complete substitute for formal statistical correction for multiple comparisons, and the risk of capitalizing on chance associations is not negligible. In the future, more rigorous statistical correction (such as Bonferroni correction or FDR control) in multi-center prospective cohorts is needed to further confirm its predictive value. Finally, external validation was lacking in this study, so further confirmation is needed to confirm whether the model can be generalized to new cohorts.

## Conclusions

We demonstrated an interpretable machine learning model for POD risk prediction in elderly patients with CHD. The machine learning model based on seven features was used to predict the occurrence of POD in elderly patients with CHD. Among them, the GBM model performed the best. Among these features, elevated CFS grade, lower MMSE score and higher AIS score significantly enhanced the predictive ability of the model. However, external validation is required before the model can be applied in clinical Settings.

## Data Availability

The raw data supporting the conclusions of this article will be made available by the authors, without undue reservation.

## Supplementary material

Supplementary Figure 1 The receiver operating characteristic curves, decision curves and calibration curves of the ten models. **(A)** ROC curve of the validation set; **(B)** Decision curve of the validation set; **(C)** Calibration curve of the validation set.
