# Supplementary material for: Machine learning-based predictive model for postoperative delirium of elderly patients with coronary heart disease undergoing non-cardiac surgery: a retrospective cohort study
Source: Front Psychiatry. 2026 Mar 27;17:1780056. doi: 10.3389/fpsyt.2026.1780056 (PMC13066171; doi:10.3389/fpsyt.2026.1780056)
Supplement: Supplementary file 1 [file Table1.docx]

glm.tune.grid = NULL

svm.tune.grid = expand.grid(sigma = c(0.1,0.01,0.001), C = c(0.1,0.5))

gbm.tune.grid = expand.grid(n.trees = 100, interaction.depth = c(2,3),shrinkage = c(0.1,0.01), n.minobsinnode = 5)

nnet.tune.grid = expand.grid(size = c(3:5),decay = 0.6)

rf.tune.grid = expand.grid(mtry = c(2:5),numRandomCuts = 3)

xgb.tune.grid = expand.grid(nrounds = 10,max_depth = c(3:5),eta = c(0.1,0.01,0.001),

gamma = 0.5,colsample_bytree = 0.5,min_child_weight = 1,subsample = 0.6)

knn.tune.grid = expand.grid(kmax = c(3:15) ,distance = 1,kernel = "optimal")

ada.tune.grid = expand.grid(mfinal = 2,maxdepth = c(2:5),coeflearn = "Zhu")

9.LightGBM

train = dev

train$Result = ifelse(train$Result=="Yes",1,0)

dtrain = lgb.Dataset(as.matrix(train[2:ncol(train)]), label = train$Result)

test = vad[,var]

test$Result = ifelse(test$Result=="Yes",1,0)

dtest = lgb.Dataset.create.valid(dtrain, as.matrix(test[2:ncol(test)]), label = test$Result)

params = list(

objective = "binary",

metric = "auc",

min_data = 1L,

learning_rate = 1.0,

num_threads = 2L,

force_col_wise = T)

valids = list(test = dtest)

lightgbm_model = lgb.train(params = params,data = dtrain,

nrounds = 5L,

valids = valids,

early_stopping_rounds = 3L)

train_probe$LightGBM = predict(lightgbm_model,newdata = as.matrix(dev[2:ncol(dev)]),type = 'prob')

test_probe$LightGBM = predict(lightgbm_model,newdata = as.matrix(vad[2:ncol(vad)]),type = 'prob')

lightGBM_Imp = lgb.importance(lightgbm_model, percentage = TRUE)

10.CatBoost

train = dev

train$Result = ifelse(train$Result=="Yes",1,0)

train <- as.data.frame(lapply(train, function(x) {

if (is.integer(x)) {

return(as.numeric(x))

}

return(x)

}))

train_pool = catboost.load_pool(as.matrix(train[2:ncol(train)]),label = train$Result)

test = vad[,var]

test$Result = ifelse(test$Result=="Yes",1,0)

test <- as.data.frame(lapply(test, function(x) {

if (is.integer(x)) {

return(as.numeric(x))

}

return(x)

}))

test_pool = catboost.load_pool(as.matrix(test[2:ncol(test)]),label = test$Result)

fit_params = list(

iterations = 100,

use_best_model = TRUE,

eval_metric = 'AUC',

ignored_features = c(4, 9),

border_count = 32,

depth = 5,

learning_rate = 0.03,

random_seed =123)

Catboost_model = catboost.train(train_pool, test_pool, fit_params)

Catboost_model

train_probe$CatBoost = catboost.predict(Catboost_model, train_pool, prediction_type = 'Probability')

test_probe$CatBoost = catboost.predict(Catboost_model, test_pool, prediction_type = 'Probability')

Catboost_Imp = catboost.get_feature_importance(Catboost_model)

Catboost_Imp = data.frame(Feature = colnames(dev)[2:ncol(dev)],Overall = Catboost_Imp)

write.csv(Catboost_Imp,"CatBoost_important.csv",row.names = F)
