# Supplementary figures and images for: Machine learning-based predictive model for postoperative delirium of elderly patients with coronary heart disease undergoing non-cardiac surgery: a retrospective cohort study
Source: Front Psychiatry. 2026 Mar 27;17:1780056. doi: 10.3389/fpsyt.2026.1780056 (PMC13066171; doi:10.3389/fpsyt.2026.1780056)

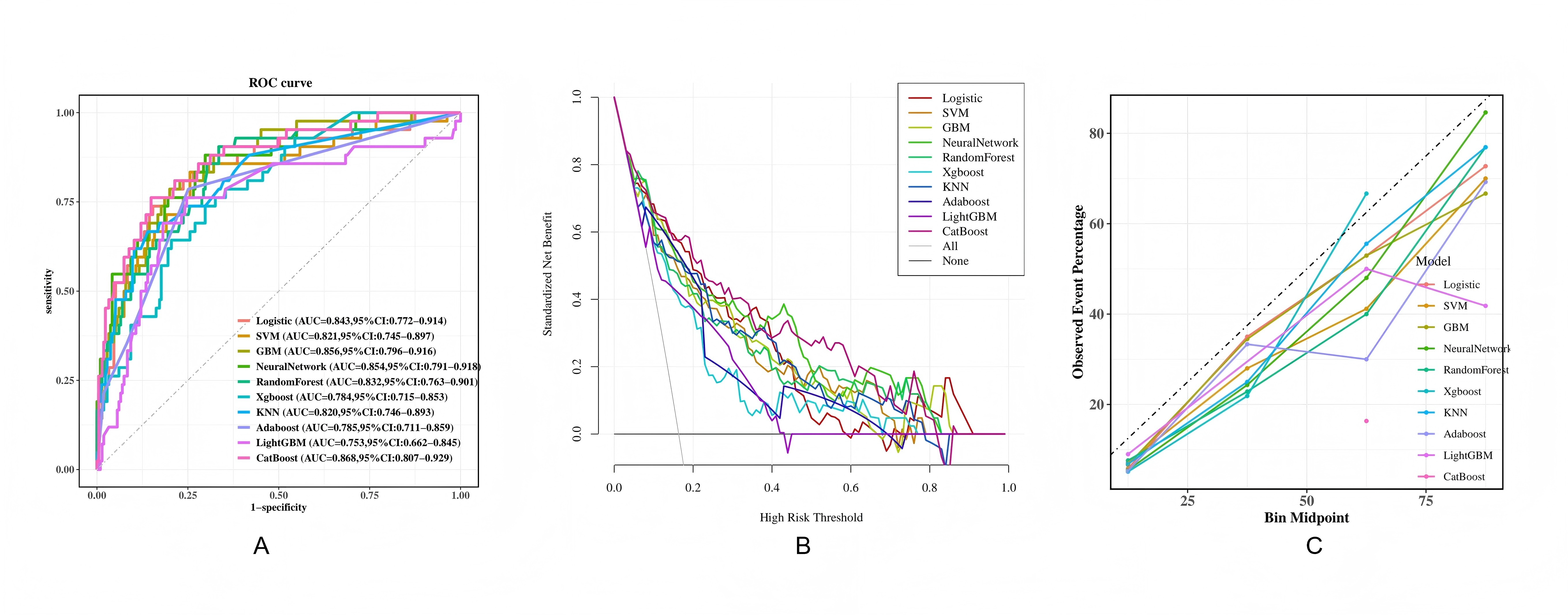

Supplement: Supplementary Figure 1 — The receiver operating characteristic curves, decision curves and calibration curves of the ten models. (A) ROC curve of the validation set; (B) Decision curve of the validation set; (C) Calibration curve of the validation set. [file Image1.jpeg]
